# 5-HT_1A_ receptor antagonism decreases motor activity and influences dopamine and serotonin metabolization pathways, primarily in cingulate cortex and striatum

**DOI:** 10.1038/s41598-025-26268-7

**Published:** 2025-11-04

**Authors:** Benedetta Fazari, Susanne Nikolaus, Owen Y. Chao, Filipe Rodrigues Almeida, Laila Abdel-Hafiz, Markus Beu, Jan Henke, Christina Antke, Eduards Mamlins, Hubertus Hautzel, Hans-Wilhelm Müller, Joseph P. Huston, Charlotte von Gall, Frederik L. Giesel

**Affiliations:** 1 Institute of Anatomy II, Medical Faculty, Heinrich-Heine University, Universitätsstr. 1, 40225 Düsseldorf, Germany; 2 Clinic of Nuclear Medicine, Medical Faculty, Heinrich-Heine University, Moorenstr. 5, 40225 Düsseldorf, Germany; 3 Graduate Institute of Brain and Mind Sciences, College of Medicine, National Taiwan University, Taipei, Taiwan; 4 Clinic for Nuclear Medicine, University Hospital Essen, University Duisburg Essen, Hufelandstraße 55, 45122 Essen, Germany; 5 Center for Behavioural Neuroscience, Institute of Experimental Psychology, Heinrich-Heine University, Universitätsstr. 1, 40225 Düsseldorf, Germany

**Keywords:** 5-HT_1A_ receptor, WAY100,635, High performance liquid chromatography, Metabolization, Neuroscience, Physiology

## Abstract

We assessed the effect of the 5-HT_1A_ receptor (R) antagonist WAY100,635 on motor behaviors, object place learning and the regional levels of dopamine (DA), serotonin (5-HT) and their metabolites in the rat brain. After a single dose of either WAY100,635 (0.4 mg/kg) or vehicle (0.9% NaCl), recognition memory was assessed together with motor/exploratory behaviors. After sacrifice, regional DA, 5-HT and metabolite levels were determined with HPLC. Overall activity and exploratory behavior were reduced by WAY100,635. Object place recognition did not differ between treatments. WAY100,635 promoted DA metabolization (1) by both monoamine oxidase (MAO) and catechol-O-methyl transferase (COMT) in cingulate, caudateputamen, thalamus and cerebellum, (2) solely by MAO in dorsal hippocampus and (3) solely by COMT in ventral hippocampus and brainstem, but suppressed DA metabolization (by both MAO and COMT) in nucleus accumbens. It promoted 5-HT metabolization (by MAO) in cingulate, caudateputamen, dorsal hippocampus and brainstem, but suppressed it in nucleus accumbens, thalamus and cerebellum. WAY100,635 altered activity and exploratory behavior as well as the quantitative relations between the neurotransmitter/metabolite levels in the individual brain regions, by inducing region-specific shifts in the metabolization pathways.

## Introduction

The serotonin (5-HT)_1A_ receptor (5-HT_1A_R) is the main inhibitory 5-HTR subtype in the mammalian brain (for review see^1^) and is implicated in disorders like major depression and anxiety. In both diseases, conflicting 5-HT_1A_R binding patterns have been reported, with reductions^2–4^ or increases^5,6^, suggesting complex and presumably region-dependent roles.

*N*-[2-[4-(2-Methoxyphenyl)-1-piperazinyl]ethyl]-*N*-2-pyridinylcyclohexanecarbox-amide maleate (WAY100,635;^7^) is a widely used 5-HT_1A_R antagonist in rodent studies. However, systemic WAY100,635 either reduced ambulation (0.4 mg/kg intraperitoneally [i.p.],^8^) or had no effect (0.3 mg/kg subcutaneously [s.c.],^9^; 0.4 mg/kg i.p.,^10–12^). Rearing was either increased (0.4–2.0 mg/kg i.p.,^8,13^) or remained unaltered (0.3 mg/kg s.c.,^9^; 0.4 mg/kg i.p.,^10–12^). Also, object recognition studies were inconsistent, with either no effect (0.3 and 1 mg/kg, i.p.,^14^) or increased exploration of a novel object (0.01—0.05 mg/kg s.c.,^15^; 1 mg/kg i.p.,^16^).

5-HT is metabolized by monoamine oxidase (MAO) to 5-hydroxyindole acetic acid (5-HIAA;^17^). DA metabolism involves MAO and catechol-0-methyltransferase (COMT), degrading DA to dihydroxyphenylacetic acid (DOPAC) and 3-methoxytyramine (3-MT), respectively^18^, which both are further converted to homovanillic acid (HVA;^19^).

Microdialysis studies in rats show that systemic WAY100,635 did not alter 5-HT concentrations in nucleus accumbens (NAC; 0.4 mg/kg intraperitoneally [i.p.],^11^), hippocampus (HIPP; 0.4 mg/kg i.p.,^11^) or frontal cortex (FC; 0.16 mg/kg subcutaneously [s.c.],^20^), but elevated 5-HIAA levels in the HIPP (0.4 mg/kg i.p.,^11^). DA findings are inconsistent: in caudateputamen (CP), DA release increased (0.1–0.5 mg/kg s.c.,^21^) or remained unchanged (0.1 mg/kg s.c.,^22^), while DA levels were neither affected in NAC (0.1, 0.2 and 0.5 mg/kg s.c., ^21^; 0.4 mg/kg i.p.,^12^; 0.16 mg/kg s.c.,^23^ nor in FC (0.16 mg/kg s.c., ^20^). Also, DOPAC levels remained unaltered (0.4 mg/kg i.p.,^12^). However, in an imaging study, WAY100,635 (0.4 mg/kg i.p) reduced D_2/3_R binding in CP, thalamus (THAL), FC, parietal cortex and ventral HIPP (vHIPP), indicating elevated DA^8^.

We previously demonstrated that network analysis can detect changes in regional neurotransmitter processing following pharmacological interventions^24^. The 5-HT_2A_R antagonist altanserin (ALT) suppressed DA metabolism in the THAL, while the agonist 2,5-dimethoxy-4-iodoamphetamine (DOI) extended this suppression to mesolimbic and nigrostriatal regions associated with the THAL. Both compounds suppressed 5-HT metabolism in THAL; additionally, DOI exerted suppression in NAC and CING, whereas ALT promoted metabolization in dorsal HIPP (dHIPP). On the basis of these findings, we hypothesized that also the 5-HT_1A_R antagonist WAY100,635 would influence DA and 5-HT release and metabolism in regions of the nigrostriatal and mesolimbic system, the direction and extent of these alterations being relevant for understanding the role of 5-HT_1A_R in neuropsychiatric disorders.

Given the inconsistent neurochemical effects of WAY100,635, we extended the investigations of its impact on DA, 5-HT, their metabolites (DOPAC, 3-MT, HVA, 5-HIAA) to further regions of the rat monoaminergic system (CING, CP, NAC, THAL, dHIPP, vHIPP, brainstem [BS], cerebellum [CER]). Building on our previous work^24^, we analyzed enzyme-specific metabolic changes and behavioral effects by means of a modified novelty preference test assessing recognition memory for object and place^25^.

## Materials and methods

### Animals

Studies were conducted on a total of 25 male Wistar rats (ZETT, Heinrich-Heine University, Düsseldorf, Germany), weighing 415 ± 28 g (mean ± standard deviation [S.D.]; age: 3 – 4 months). All rats underwent both behavioral and neurochemical measurements (WAY100,635: n = 12, SAL: n = 13). From the analysis of neurochemical findings, however, those data were excluded, which exceeded the respective group means by more than twice the S.D. Animals were randomly assigned to the treatment groups.

Rats were maintained in standard makrolon cages (59 × 38 × 20 cm, 3 animals per cage) in a climate cabinet (Scantainer, Scanbur BK, Karslunde, Denmark; temperature: 20° C, air humidity: 70%) with an artificial light–dark cycle (lights on at 6:00 a.m., lights off at 6:00 p.m.) and food and water freely available. The study was approved by the regional authority (reference number of ethical approval: AZ 81–02.04.2017.A450; Landesamt für Natur, Umwelt und Verbraucherschutz, Nordrhein-Westfalen, Recklinghausen, Germany) and carried out in accordance with the European Communities Council Directive (86/609/EEC), the German Law on the Protection of Animals and ARRIVE guidelines.

### Drug treatment

Rats received either WAY100,635 (Sigma-Aldrich, Taufkirchen, Germany; dissociation constant [K_d_]: 0.28 nM,^26^, molecular weight: 538.64 g/mol; dose: 0.4 mg/kg i.p., injection volume: 1 ml/kg, concentration: 0.4 mg/ml) or isotonic saline (SAL; B. Braun Melsungen AG, Melsungen, Germany; molecular weight: 58.5 g/mol; dose: 1 ml/kg i.p.). The dose of 0.4 mg/kg was chosen based on our previous study^8^, which had shown effects of WAY100,635 on DA levels in a variety of regions including CP, THAL, FC, parietal cortex and vHIPP. Moreover, the dose of 0.4 mg/kg was previously shown to block cocaine-induced increases of locomotion^10–12^.

### Rat behavior

Recognition memory for object and place (for review see^27^) was assessed together with motor/exploratory behaviors as previously described (see, e.g.,^28^). On two consecutive days, the rats were placed for 10 min into an open field (PhenoTyper®, Noldus Information Technology, Wageningen, Netherlands; dimensions: 45 × 45 × 56 cm, illumination: 19 lx) for the purpose of habituation. One day later, they were first subjected to a 5-min sample trial and a 5-min test trial with two identical polyhedrons (material: lead, length: 9.5 cm, width: 7 cm, height: 4 cm; color: green) located opposite to each other at the middle of the left and right side of the apparatus at a distance of 5 cm to the respective wall. After completion of the sample trial, the rats were injected WAY100,635 or SAL. Thirty min post-challenge, a 5-min test trial was performed with one of the polyhedrons moved to the middle of the back side of the apparatus and the other polyhedron replaced by a cylinder (material: lead, diameter: 5.6 cm, height: 8 cm; color: grey). Placement of the cylinder at the left and the right side of the apparatus was counterbalanced. Duration (sec) and frequency (n) of object exploration in sample- and test-trial were determined by registering physical contact with snout or fore-paws. Increased exploration of the novel object (cylinder) in the test trial indicates preferential memory for *what*, while increased exploration of the displaced object (polyhedron) indicates preferential memory for *where*^25^. Evaluation was performed with EthoVision® XT (Noldus Information Technology, Wageningen, Netherlands) with the experimenter (S.N.) blinded to the precedent treatment.

In the test trial, also durations (sec) and frequencies (n) of the following behavioral parameters were registered: (1) ambulation (as measure of motor activity), (b) sitting, (c) grooming, (d) rearing freely and against the walls of the open field (as a general measure of active exploration not related to the objects), (e) explorative movements of head, neck and shoulders in the open field (also not related to the objects). Furthermore, based on the movement of the animal’s center point, EthoVision XT automatically determined the distance in cm covered by the rat.

Behavioral tests were performed on all rats after either WAY100,635 or SAL. The behavioral results obtained after WAY100,635 have been previously published in relation to regional DA transporter binding^29^. Behavioral studies were conducted in the light phase between 9:00 a.m. and 5:00 p.m.

### Neurochemistry

After completion of the test trial (35 min post-challenge), animals were sacrificed with an overdose of pentobarbital natrium (Narcoren®, Boehringer Ingelheim Pharma GmbH & Co.KG, Ingelheim am Rhein, Germany, concentration: 0.16 g/ml, dose: 2.5 ml/kg i.p.). Subsequently, rats were decapitated and the brains taken out. From both hemispheres, CING, CP, NAC, THAL, dHIPP, vHIPP, BS and CER were dissected. Tissue samples were homogenized with an ultrasonic cell disruptor (Microsom™, Misonix, Farmingdale, USA) in 500 ml of 0.05 M perchloric acid (Sigma-Aldrich, Taufkirchen, Germany) and centrifuged at 12,000 revolutions/min (rpm) for 20 min at 4 °C (Eppendorf Centrifuge 5417 R, Hamburg, Germany). Subsequently, the samples were filtered by centrifugation at 2000 rpm for 2 min at 4 °C and stored at -80 °C until analysis.

The levels of DA, 5-HT, the DA metabolites DOPAC, 3-MT and HVA and the 5-HT metabolite 5-HIAA were determined with high performance liquid chromatography (HPLC). Monoamines were analyzed, using a Nucleosil 120-5C18 column (Macherey & Nagel, Düren, Germany). The mobile phase consisted of the following compounds dissolved in 1 l of bidistilled water: 14.14 g chloroacetic acid (0.15 M; Sigma-Aldrich, Taufkirchen, Germany), 4.66 g sodium hydroxide (0.12 M; Mallinckrodt Baker Inc, Phillipsburg, USA), 0.30 g potassium chloride (4 mM; Merck, Darmstadt, Germany), 0.20 g sodium octylsulfate (0.86 mM; Sigma-Aldrich, Taufkirchen, Germany), 0.25 g ethylenediaminetetraacetic acid (0.67 mM; VWR International GmbH, Darmstadt, Germany), 35 ml acetonitrile (0.9 mM; VWR International GmbH, Darmstadt, Germany) and 18 ml tetrahydrofuran (2 mM; VWR International GmbH, Darmstadt, Germany). The pH was adjusted to 3.0 by adding either chloracetic acid or sodium hydroxide.

The electrochemical detector (DECADE Elite, Antec Scientific, Leiden, The Netherlands) was set at a potential of 530 mV versus an Ag/AgCl (ISAAC) reference electrode (temperature: 35 °C, range: 10 nA). Neurotransmitter and metabolite levels were analyzed with Chrom Perfect Software (Chrom Perfect Version 5.5.6, Justice Laboratory Software, Denville, USA).

### Statistical analysis

*Motor/exploratory behaviors and recognition memory for object and place*. Each parameter in the test trial of each treatment group was tested for normality of distribution with the Kolmogorov–Smirnov test. With the exception of grooming duration and frequency, and both duration and frequency of polyhedron exploration (p < 0.005), all parameters were normally distributed (0.087 ≤ p ≤ 0.830). If normally distributed, parameters were compared between groups with independent t tests (α = 0.05), whereas not-normally distributed parameters were compared with Mann–Whitney rank sum tests (α = 0.05).

*Neurochemistry*. Means and S.D.’s of transmitter and metabolite concentrations (pg/mg tissue, wet weight) in each brain region were computed for each treatment group. Moreover, for each treatment group the means and S.D.’s of the following turnover ratios in each brain region were computed: DOPAC/DA, 3-MT/DA, HVA/DOPAC, HVA/3-MT, 5-HIAA/5-HT.

For each compound and turnover ratio, two-way ANOVAS were calculated for the factors “treatment” and “brain region” as well as for their interaction (“treatment x brain region”). Pairwise multiple comparisons (Holm-Sidak method, overall α = 0.05) were performed separately for the factors “treatment” and “brain region” as well as for “treatment” with respect to the individual regions and for “brain region” with respect to the individual treatments.

Statistic calculations were performed with SigmaStat (version 3.5, Systat Software Inc., Erkrath, Germany).

*Correlation analysis.* The association between neurotransmitter concentrations and behavioral parameters (overall activity, duration and frequency of polyeder and cylinder exploration, ambulation, sitting, rearing, head-shoulder motility, grooming) was assessed by computing Pearson product moment correlation coefficients (r). Calculations were performed with SigmaStat (version 3.5, Systat Software Inc., Erkrath, Germany).

*Network analysis*. With this mode of analysis, the network structure of variables  - pre-defined so-called “nodes” - can be analysed by estimating path coefficients, which describe the direction (positive or negative) and the “strength” of the individual connections. Thereby, firstly, covariance matrices were estimated with gaussian graphical models employing graphical L_1_ (lasso) regularized regression in order to decrease matrix sparsity^30^, thus increasing the number of pairwise interactions. Covariance is defined as the mean of multiplication of corresponding S.D.’s of a pair of variables, and, consequently, indicates the direction of the linear relationship between these variables. By dividing the covariances of each pair of variables by the product of their S.D.’s, standardized path correlation coefficients (between -1 and + 1) are obtained, which, thus, indicate the strength in addition to the direction of their association. Here, we separately assessed the associations between the concentrations of neurotransmitter and metabolites in the individual brain regions after treatment with either WAY100,635 or SAL. Network analyses were computed with JASP (version 0.18.1, JASP Team*,* © 2023 University of Amsterdam).

## Results

### Motor/exploratory behaviors and object recognition

**Fig. 1:**
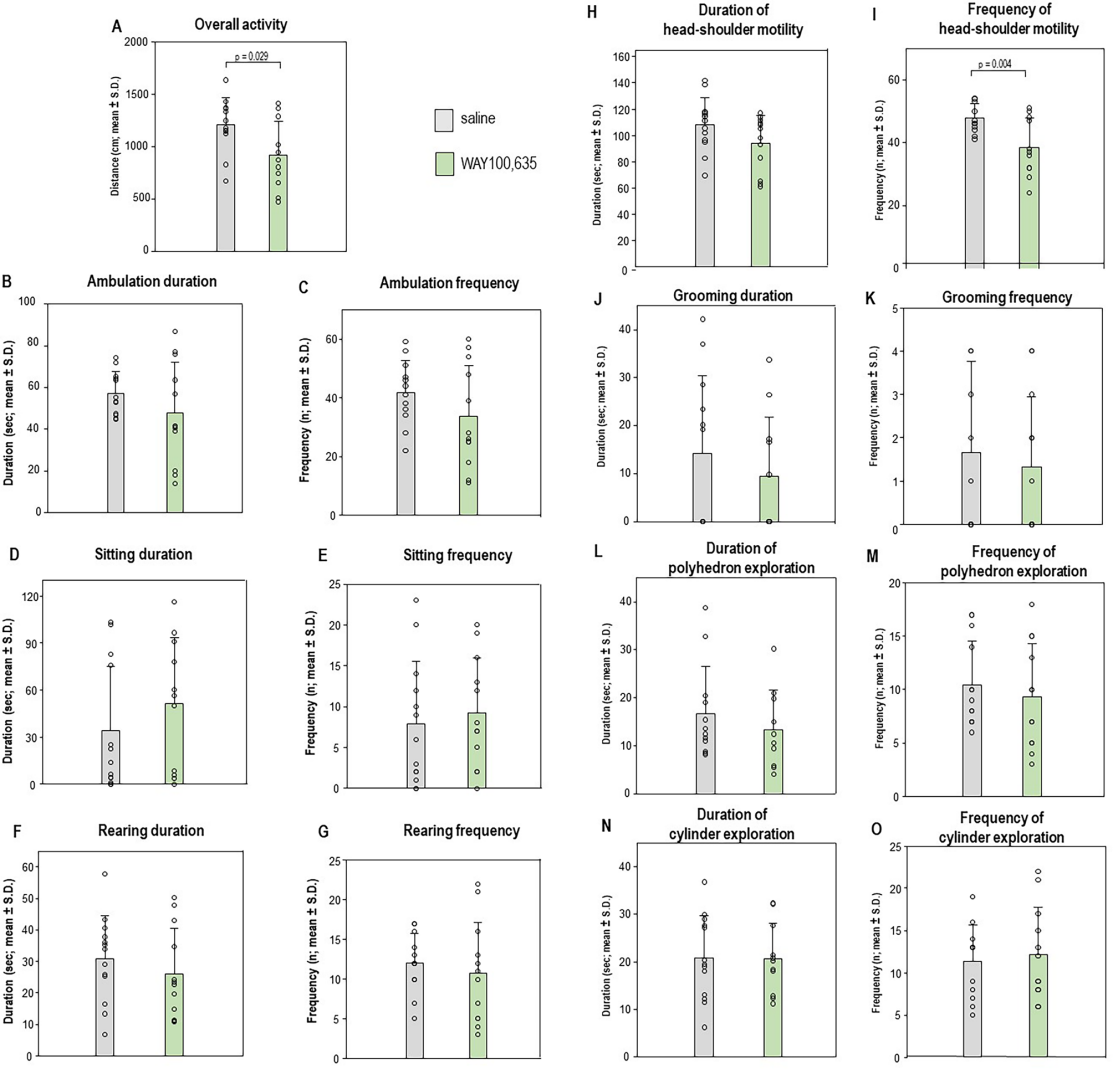
Overall activity (**A**), ambulation duration (**B**), ambulation frequency (**C**), sittíng duration (**D**), sitting frequency (**E**), rearing duration (**F**), rearing frequency (**G**), duration of head-shoulder motility (**H**), frequency of head-shoulder motility (**I**), grooming duration (**J**), grooming frequency (**K**), duration of polyhedron exploration (**L**), frequency of polyhedron exploration (**M**), duration of cylinder exploration (**N**) and frequency of cylinder exploration (**O**) after challenge with saline (SAL; 0.9% solution; 1 ml/kg i.p.) and WAY100,635 (0.4 mg/kg i.p.). Given are mean values and standard deviations (S.D.). The significant p values given in the figure were obtained with independent t tests (α ≤ 0.05).

Overall activity (t, 3.252, p = 0.029) and frequency of head-shoulder motility (t, 3.204, p = 0.004) were reduced after WAY100,635 relative to SAL (Fig. 1A and I). All other parameters were not different between groups (0.0331 ≤ t ≤ 1.747, 0.094 ≤ p ≤ 0.974; see Figs. 1B-H and Figs. J-O).

### Neurochemistry

Neurochemical results are given in Table 1 and 2 as well as in Figs. 2 and 3. Results of network analyses are given in Figs. 4,5,6,7,8,9 and 10.

**Table 1:** Concentrations (pg/mg brain tissue, wet weight; means ± standard deviations [S.D.s]) of dopamine (DA), serotonin (5-HT) and metabolites (3,4-dihydroxyphenylacetic acid [DOPAC], 3-methoxytyramine [3-MT], homovanillic acid [HVA], 5-hydroxyindole-acetic acid [5-HIAA]) in the individual brain regions (cingulate [CING], caudateputamen [CP], nucleus accumbens [NAC], thalamus [THAL], dorsal hippocampus [dHIPP], ventral hippocampus [vHIPP], brainstem [BS], cerebellum [CER]) after treatment with saline (SAL), and WAY100,635 as obtained with high performance liquid chromatography.

Region	Treatment	DA	DOPAC	3-MT	HVA	5-HT	5-HIAA
CING	SAL WAY100,635	23.09 ± 19.82 40.37 ± 30.25	2390.00 ± 1721.85 2571.77 ± 1381.85	233.26 ± 157.11 258.91 ± 179.70	291.54 ± 251.18 513.91 ± 389.38	11.10 ± 6.39 7.27 ± 5.71	753.09 ± 369.09 492.06 ± 303.73
CP	SAL WAY100,635	58.03 ± 43.47 187.13 ± 145.76	680.76 ± 543.53 384.25 ± 180.35	11.72 ± 6.87 30.40 ± 3.47	151.38 ± 114.00 140.98 ± 40.51	2.30 ± 1.14 3.99 ± 3.37	53.51 ± 28.62 67.87 ± 18.83
NAC	SAL WAY100,635	91.12 ± 78.53 150.91 ± 83.07	296.88 ± 171.09 504.36 ± 521.49	20.19 ± 17.01 16.22 ± 8.54	188.96 ± 218.23 165.19 ± 127.45	10.00 ± 8.09 12.22 ± 8.90	99.99 ± 43.28 81.76 ± 27.09
THAL	SAL WAY100,635	171.32 ± 94.99 52.49 ± 34.55	79.96 ± 37.56 32.49 ± 17.71	8.41 ± 4.31 12.16 ± 8.68	10.21 ± 5.05 6.16 ± 2.62	49.02 ± 25.34 31.45 ± 15.74	304.55 ± 91.38 161.38 ± 65.00
dHIPP	SAL WAY100,635	41.08 ± 18.35 36.54 ± 22.28	215.76 ± 133.54 172.79 ± 83.26	62.68 ± 23.64 61.99 ± 38.80	312.98 ± 170.29 268.77 ± 88.03	7.21 ± 5.13 7.31 ± 7.63	187.64 ± 11.45 181.40 ± 73.40
vHIPP	SAL WAY100,635	54.81 ± 26.41 49.14 ± 18.12	94.10 ± 60.82 65.12 ± 32.11	93.23 ± 56.96 69.10 ± 26.46	301.07 ± 124.39 178.72 ± 41.20	15.37 ± 8.76 18.80 ± 15.08	154.52 ± 86.64 111.57 ± 29.76
BS	SAL WAY100,635	91.89 ± 17.34 89.67 ± 22.19	27.56 ± 7.63 24.13 ± 6.62	172.33 ± 106.61 185.27 ± 107.22	698.79 ± 272.98 709.09 ± 259.72	145.85 ± 57.90 126.51 ± 44.31	242.20 ± 35.60 276.40 ± 79.39
CER	SAL WAY100,635	15.45 ± 7.38 10.78 ± 2.68	16.84 ± 7.17 14.92 ± 5.53	279.03 ± 101.42 273.54 ± 64.98	117.80 ± 58.06 60.18 ± 19.42	17.54 ± 10.43 14.24 ± 5.61	47.44 ± 13.28 39.43 ± 12.11

**Table 2:** Turnover ratios (means ± standard deviations [S.D.s]) of dopamine (DA) and serotonin (5-HT). Given are the ratios DOPAC/DA, 3-MT/DA, HVA/DOPAC, HVA/3-MT and 5-HIAA/5-HT in the individual brain regions (cingulate [CING], caudateputamen [CP], nucleus accumbens [NAC], thalamus [THAL], dorsal hippocampus [dHIPP], ventral hippocampus [vHIPP], brainstem [BS], cerebellum [CER]) after treatment with saline (SAL) and WAY100,635.

Region	Treatment	DOPAC/DA	3-MT/DA	HVA/DOPAC	HVA/3-MT	5-HIAA/5-HT
CING	SAL WAY100,635	258.46 ± 369.71 90.48 ± 54.40	17.50 ± 15.33 9.34 ± 8.12	0.18 ± 0.19 0.17 ± 0.11	1.58 ± 1.53 2.67 ± 3.09	89.38 ± 58.88 101.66 ± 77.34
CP	SAL WAY100,635	10.17 ± 9.38 3.08 ± 2.43	0.29 ± 0.26 0.25 ± 0.32	0.27 ± 0.07 0.37 ± 0.09	13.37 ± 7.27 7.43 ± 6.01	20.58 ± 13.30 32.09 ± 32.79
NAC	SAL WAY100,635	5.88 ± 3.68 3.04 ± 2.04	0.28 ± 0.28 0.11 ± 0.07	0.55 ± 0.84 0.37 ± 0.11	9.96 ± 0.84 12.19 ± 0.11	10.21 ± 4.22 7.84 ± 5.25
THAL	SAL WAY100,635	0.62 ± 0.36 0.74 ± 0.55	0.08 ± 0.08 0.27 ± 0.23	0.14 ± 0.09 0.15 ± 0.05	1.41 ± 0.99 0.73 ± 0.37	8.79 ± 6.38 7.12 ± 3.94
dHIPP	SAL WAY100,635	5.26 ± 2.31 6.28 ± 3.71	1.78 ± 0.85 1.92 ± 0.89	1.45 ± 0.38 2.14 ± 1.74	4.91 ± 3.0 8 5.68 ± 3.01	33.25 ± 13.83 42.60 ± 27.37
vHIPP	SAL WAY100,635	1.93 ± 1.06 1.30 ± 0.79	1.60 ± 1.06 1.59 ± 0.68	4.09 ± 2.13 2.90 ± 1.28	3.63 ± 2.03 2.56 ± 0.58	15.48 ± 14.13 9.97 ± 10.99
BS	SAL WAY100,635	0.32 ± 0.15 0.28 ± 0.10	2.18 ± 1.94 2.21 ± 1.60	28.38 ± 18.03 28.62 ± 9.96	5.25 ± 3.80 5.09 ± 3.35	1.93 ± 0.65 2.75 ± 2.40
CER	SAL WAY100,635	1.29 ± 0.83 1.38 ± 0.39	19.14 ± 5.90 24.51 ± 6.48	7.35 ± 3.23 4.42 ± 1.13	0.45 ± 0.21 0.21 ± 0.08	3.47 ± 0.99 3.17 ± 0.97

**Fig. 2:**
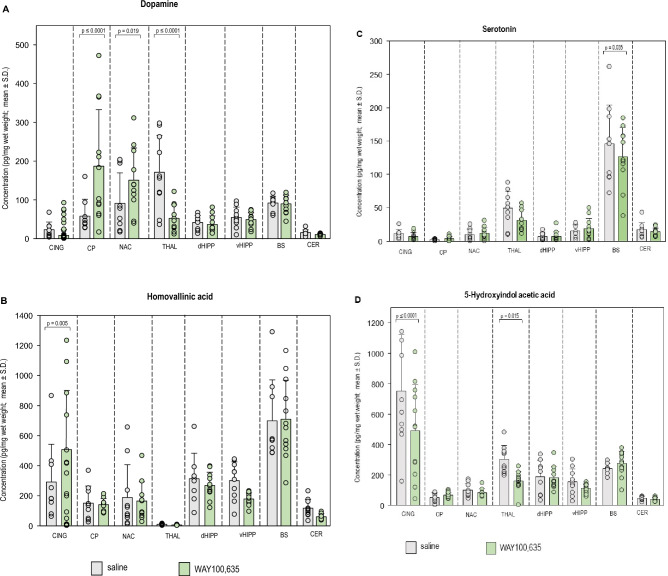
Concentrations of (**A**) dopamine (DA), (**B**) homovanillic acid (HVA). (**C**) serotonin (5-HT) and (**D**) 5-hydroxyindole-acetic acid (5-HIAA) in cingulate (CING), caudateputamen (CP), nucleus accumbens (NAC), thalamus (THAL), dorsal hippocampus (dHIPP), ventral hippocampus (vHIPP) and cerebellum after challenge with saline (SAL; 0.9% solution; 1 ml/kg and WAY100,635 (0.4 mg/kg i.p.). Given are mean values and standard deviations (S.D.). The circles represent the individual rats. The significant p values given in the figure were obtained with two-way ANOVAS and post hoc Holm-Sidak tests (α ≤ 0.05).

**Fig. 3:**
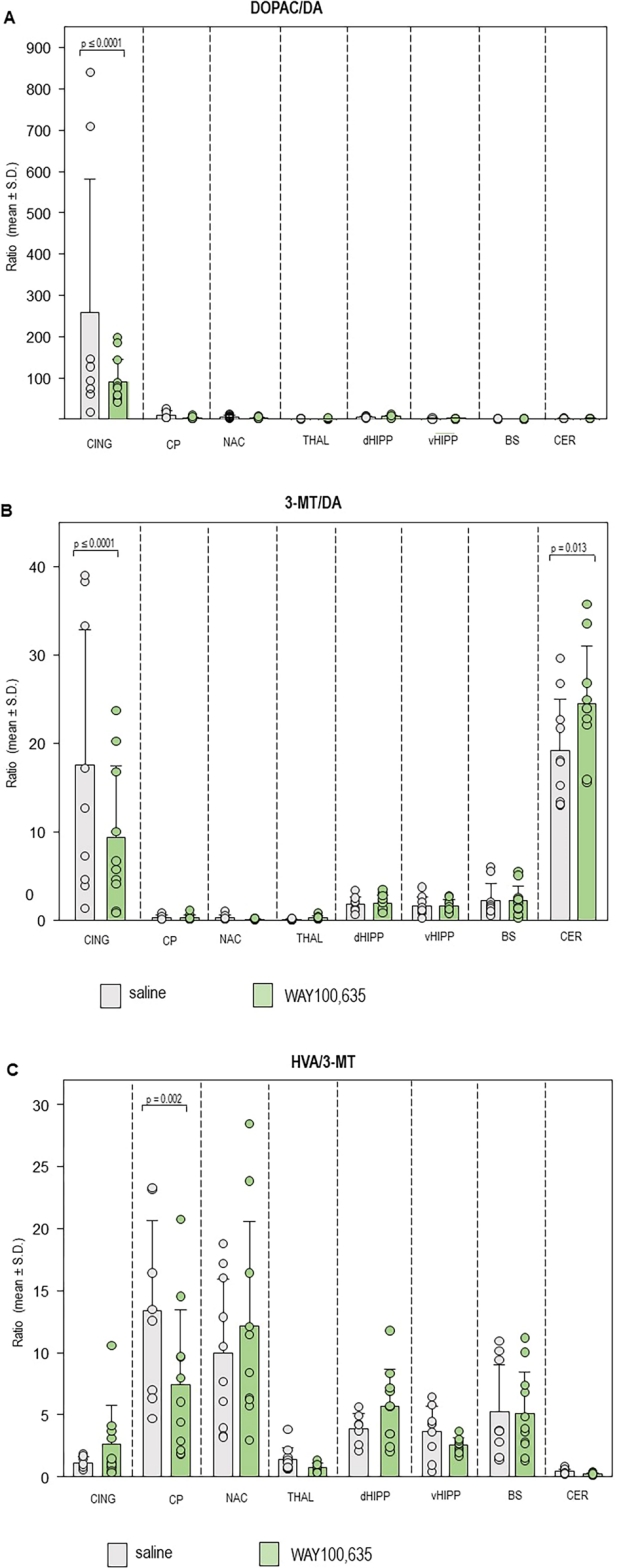
(**A**) DOPAC/DA, (**B**) 3-MT/DA and (**C**) HVA/3-MT turnover ratios in cingulate (CING), caudateputamen (CP), nucleus accumbens (NAC), thalamus (THAL), dorsal hippocampus (dHIPP), ventral hippocampus (vHIPP) and cerebellum after challenge with saline (SAL; 0.9% solution; 1 ml/kg and WAY100,635 (0.4 mg/kg i.p.). Given are mean values and standard deviations (S.D.). The circles represent the individual rats. The significant p values given in the figure were obtained with two-way ANOVAS and post hoc Holm-Sidak tests (α ≤ 0.05).

**Fig. 4:**
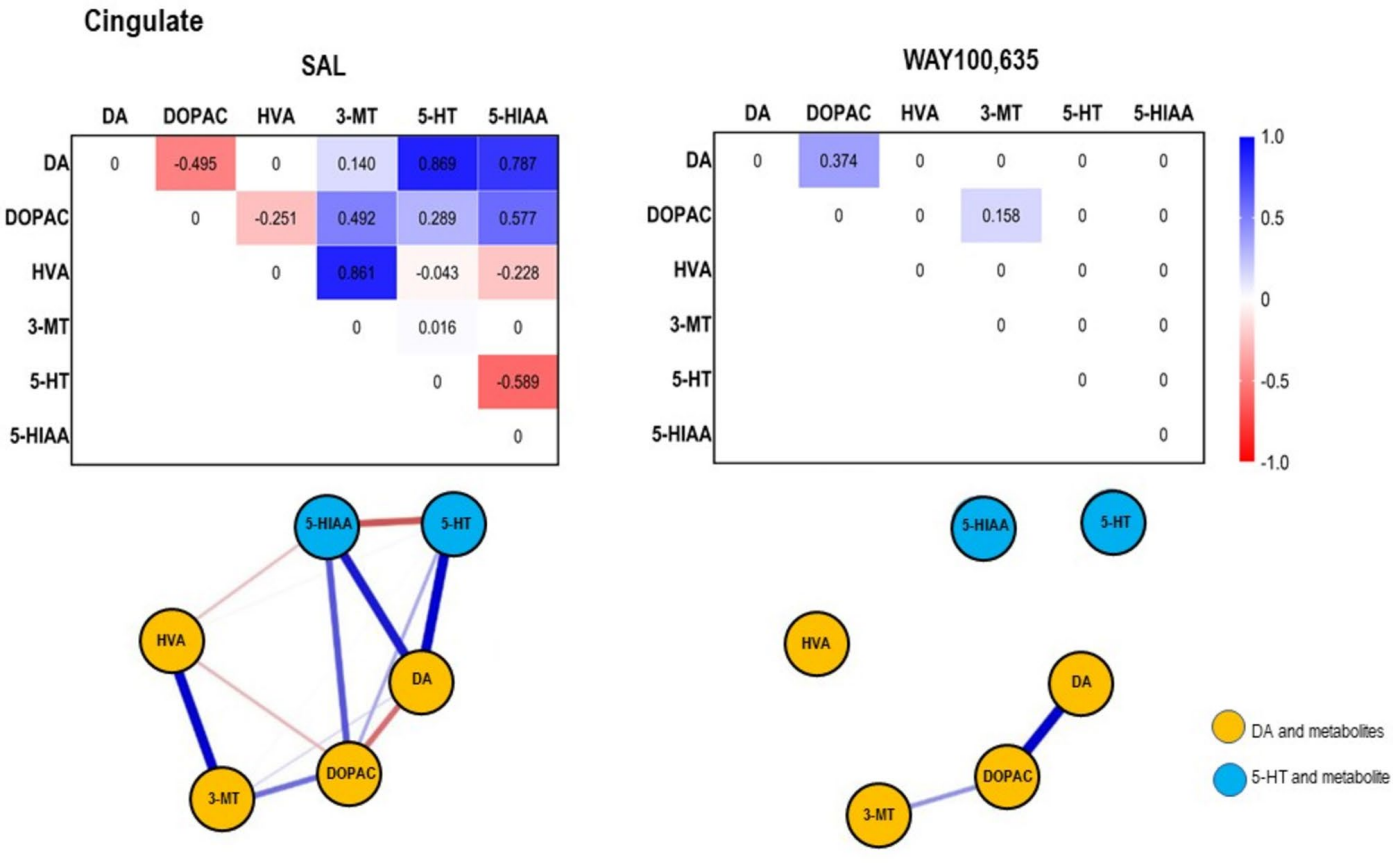
*Top*: Path coefficient matrix obtained obtained for the **cingulate** with EBICglasso modelling of neurotransmitter (DA, 5-HT) and metabolite (DOPAC, HVA, 3-MT, 5-HIAA) concentrations after treatment with SAL (13 out of 15 possible connections, sparsity: 0.133) and WAY100,635 (2 out of 15 possible connections, sparsity: 0.867). *Bottom*: Connections between DA, 5-HT and metabolites (DOPAC, 3-MT, HVA, 5-HIAA) in **cingulate** after SAL, and WAY100,635. Positive and negative associations are represented by blue and red lines, respectively. The thickness of the lines indicates the strength of the individual connections.

**Fig. 5:**
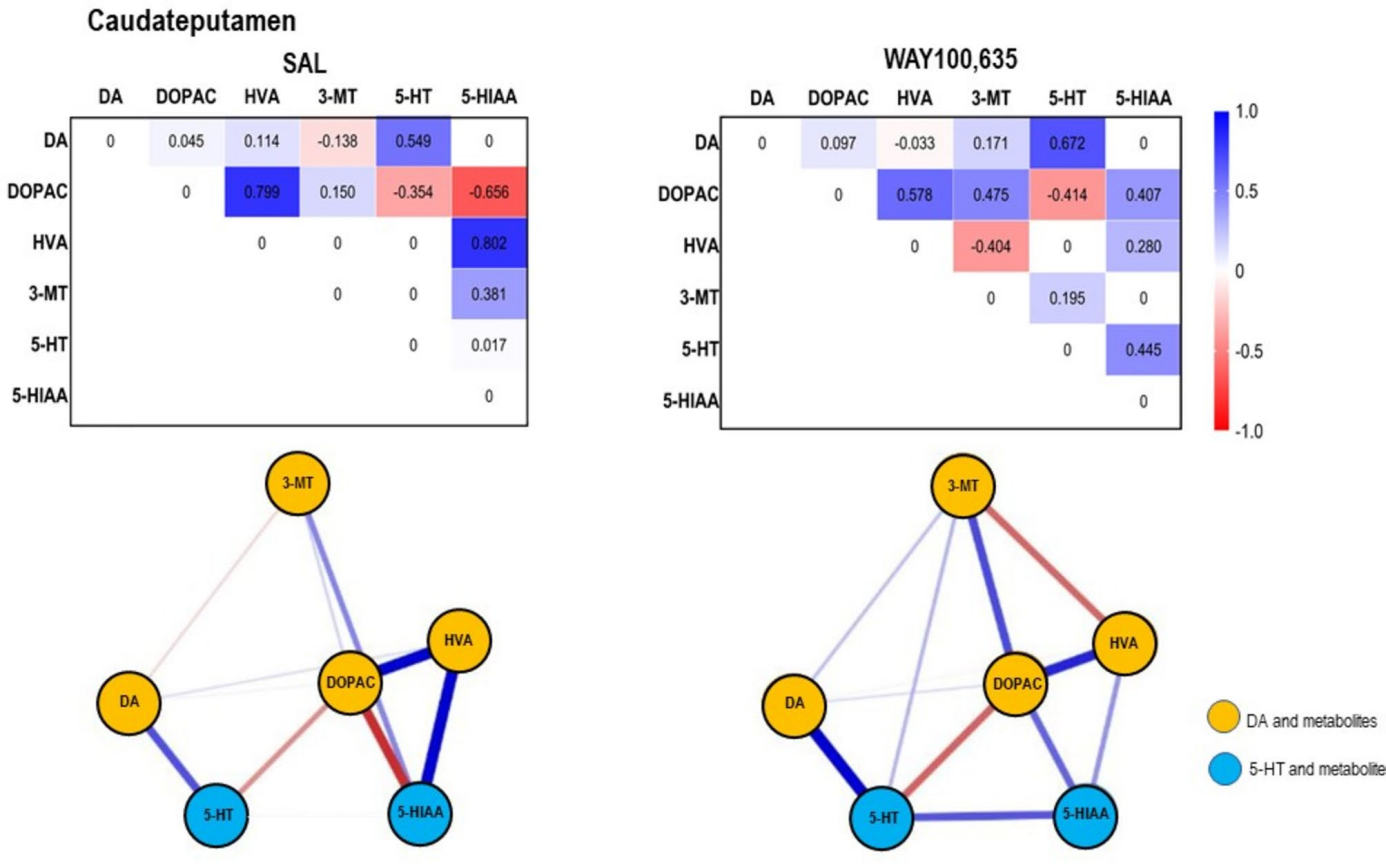
*Top*: Path coefficient matrix obtained obtained for the **caudateputamen** with EBICglasso modelling of neurotransmitter (DA, 5-HT) and metabolite (DOPAC, HVA, 3-MT, 5-HIAA) concentrations after treatment with SAL (11 out of 15 possible connections, sparsity: 0.267) and WAY100,635 (12 out of 15 possible connections, sparsity: 0.200). *Bottom*: Connections between DA, 5-HT and metabolites (DOPAC, 3-MT, HVA, 5-HIAA) in **cingulate** after SAL and WAY100,635. Positive and negative associations are represented by blue and red lines, respectively. The thickness of the lines indicates the strength of the individual connections.

**Fig. 6:**
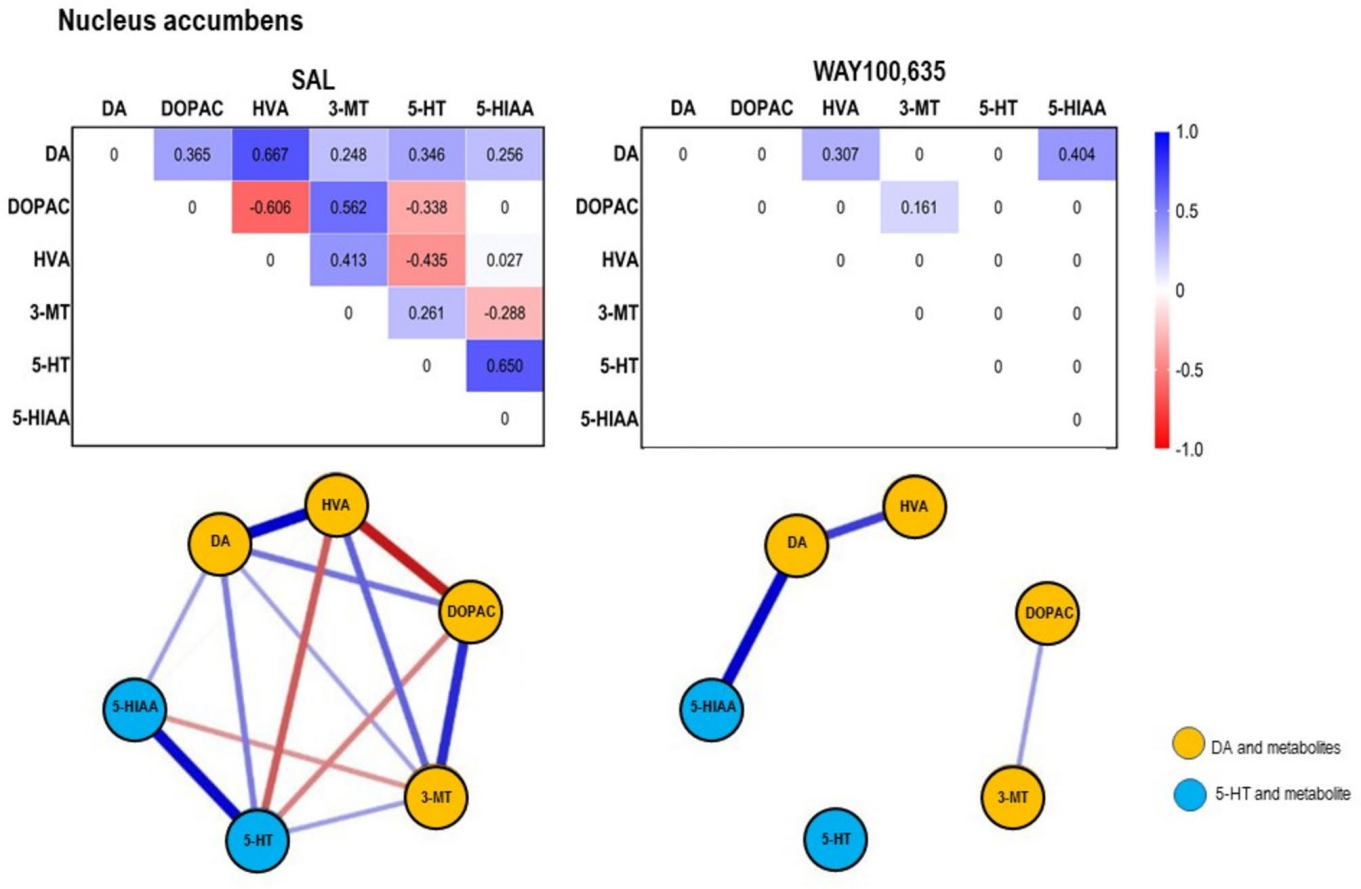
*Top*: Path coefficient matrix obtained obtained for the **nucleus accumbens** with EBICglasso modelling of neurotransmitter (DA, 5-HT) and metabolite (DOPAC, HVA, 3-MT, 5-HIAA) concentrations after treatment with SAL (14 out of 15 possible connections, sparsity: 0.067) and WAY100,635 (3 out of 15 possible connections, sparsity: 0.800). *Bottom*: Connections between DA, 5-HT and metabolites (DOPAC, 3-MT, HVA, 5-HIAA) in **nucleus accumbens** after SAL and WAY100,635. Positive and negative associations are represented by blue and red lines, respectively. The thickness of the lines indicates the strength of the individual connections.

**Fig. 7:**
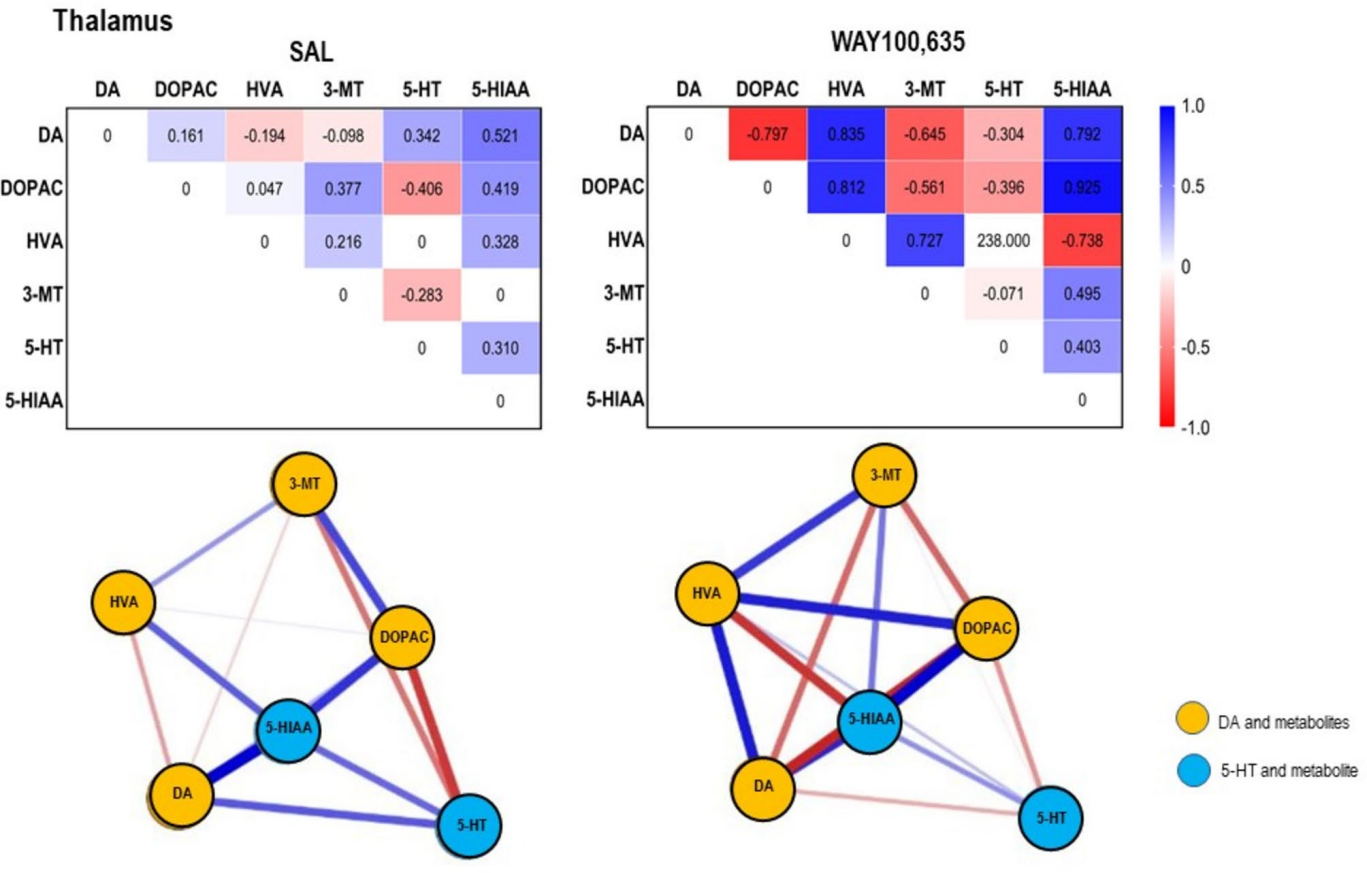
*Top*: Path coefficient matrix obtained obtained for the **thalamus** with EBICglasso modelling of neurotransmitter (DA, 5-HT) and metabolite (DOPAC, HVA, 3-MT, 5-HIAA) concentrations after treatment with SAL (13 out of 15 possible connections, sparsity: 0.133) and WAY100,635 (15 out of 15 possible connections, sparsity: 0.000). *Bottom*: Connections between DA, 5-HT and metabolites (DOPAC, 3-MT, HVA, 5-HIAA) in **thalamus** after SALand WAY100,635. Positive and negative associations are represented by blue and red lines, respectively. The thickness of the lines indicates the strength of the individual connections.

**Fig. 8:**
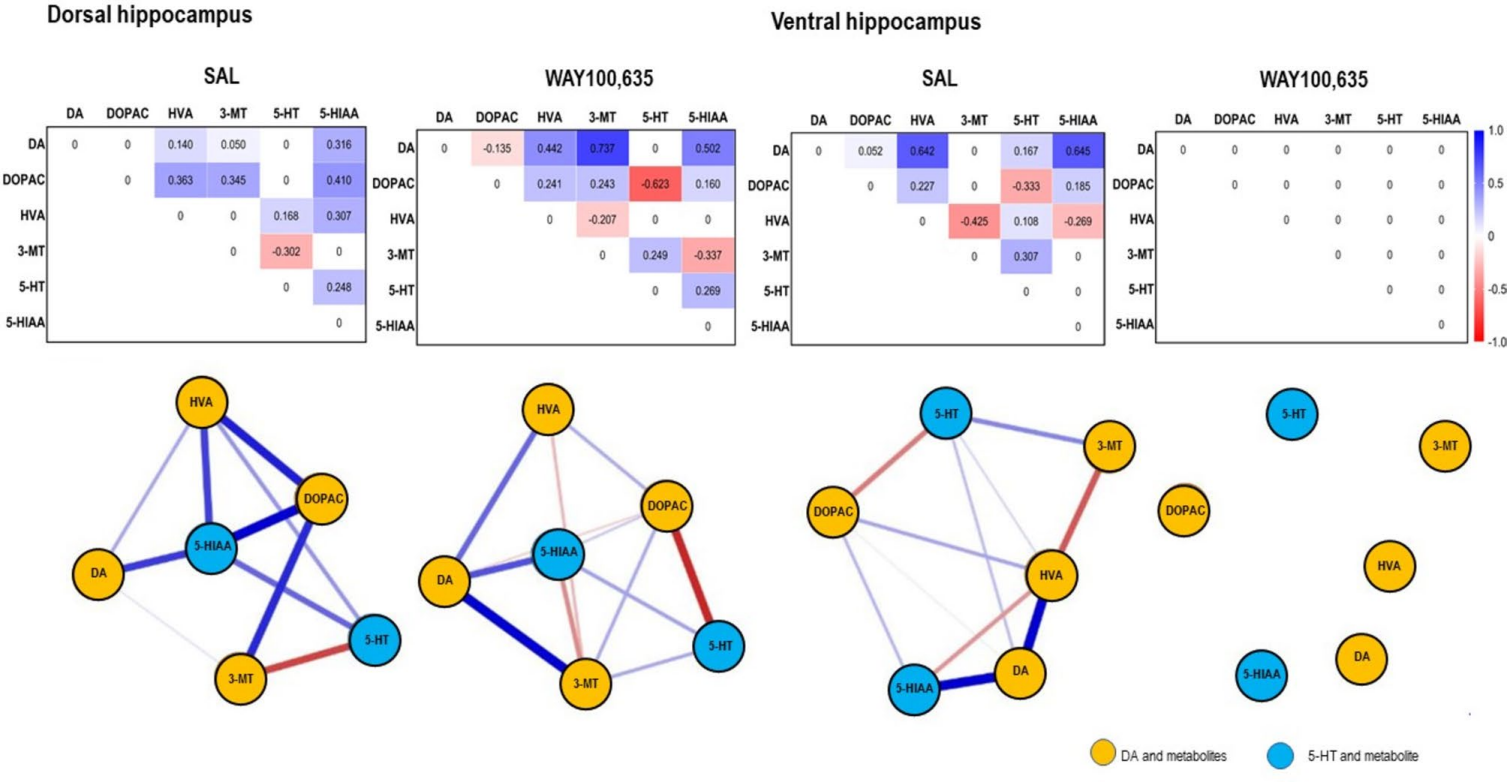
*Top*: Path coefficient matrix obtained for **dorsal and ventral hippocampus** with EBICglasso modelling of neurotransmitter (DA, 5-HT) and metabolite (DOPAC, HVA, 3-MT, 5-HIAA) concentrations after treatment with SAL (dorsal hippocampus: 10 out of 15 possible connections, sparsity: 0.333; ventral hippocampus: 11 out of 15 connections, sparsity: 0.267) and WAY100,635 (dorsal hippocampus: 12 out of 15 possible connections, sparsity: 0.200; ventral hippocampus: 0 out of 15 connections, sparsity: 1.00). *Bottom*: Connections between DA, 5-HT and metabolites (DOPAC, 3-MT, HVA, 5-HIAA) in **dorsal and ventral hippocampus** after SAL and WAY100,635. Positive and negative associations are represented by blue and red lines, respectively. The thickness of the lines indicates the strength of the individual connections.

**Fig. 9:**
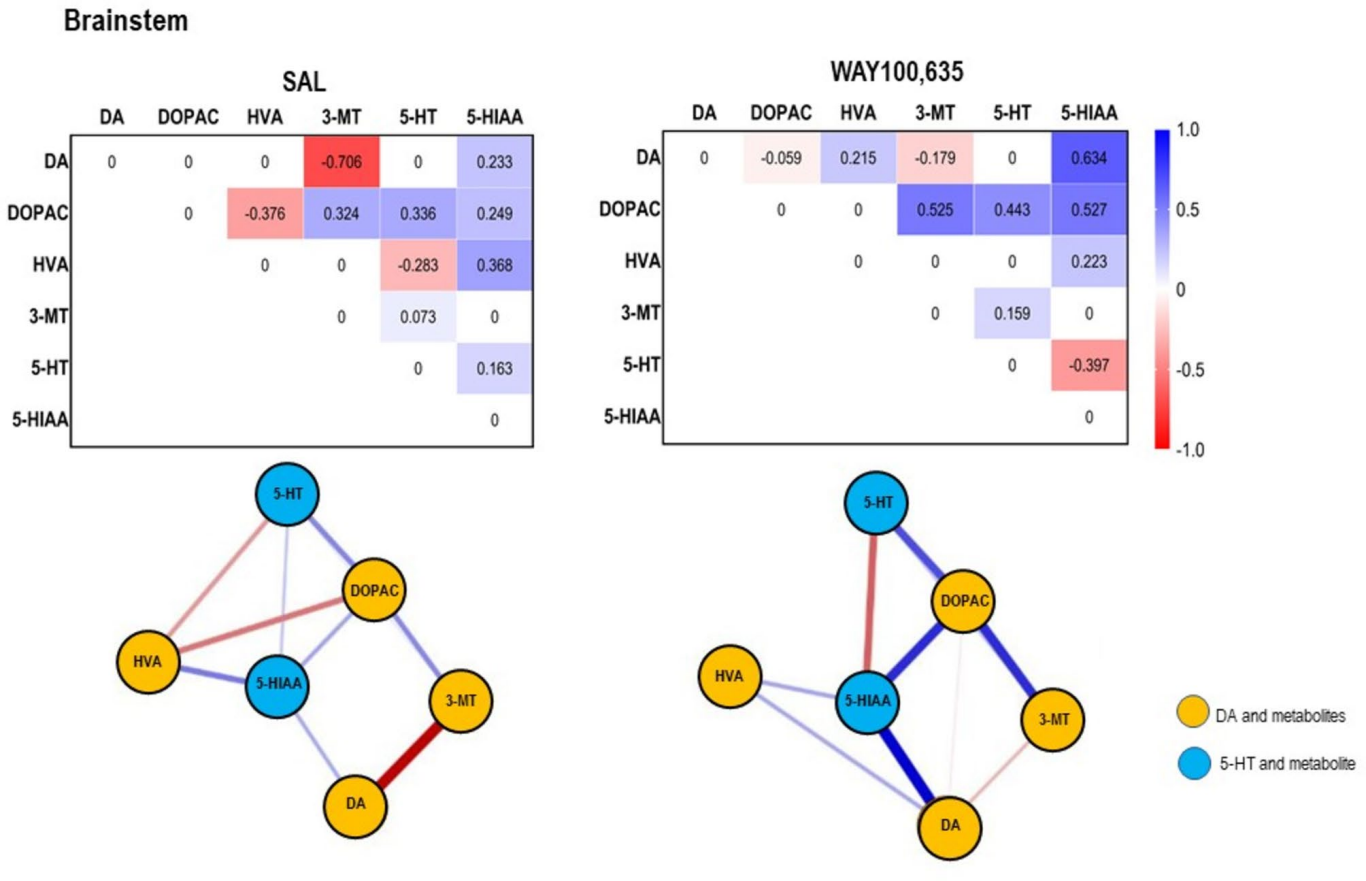
*Top*: Path coefficient matrix obtained for the **brainstem** with EBICglasso modelling of neurotransmitter (DA, 5-HT) and metabolite (DOPAC, HVA, 3-MT, 5-HIAA) concentrations after treatment with SAL (10 out of 15 possible connections, sparsity: 0.333) and WAY100,635 (10 out of 15 possible connections, sparsity: 0.333). *Bottom*: Connections between DA, 5-HT and metabolites (DOPAC, 3-MT, HVA, 5-HIAA) in **brainstem** after SAL and WAY100,635. Positive and negative associations are represented by blue and red lines, respectively. The thickness of the lines indicates the strength of the individual connections.

**Fig. 10:**
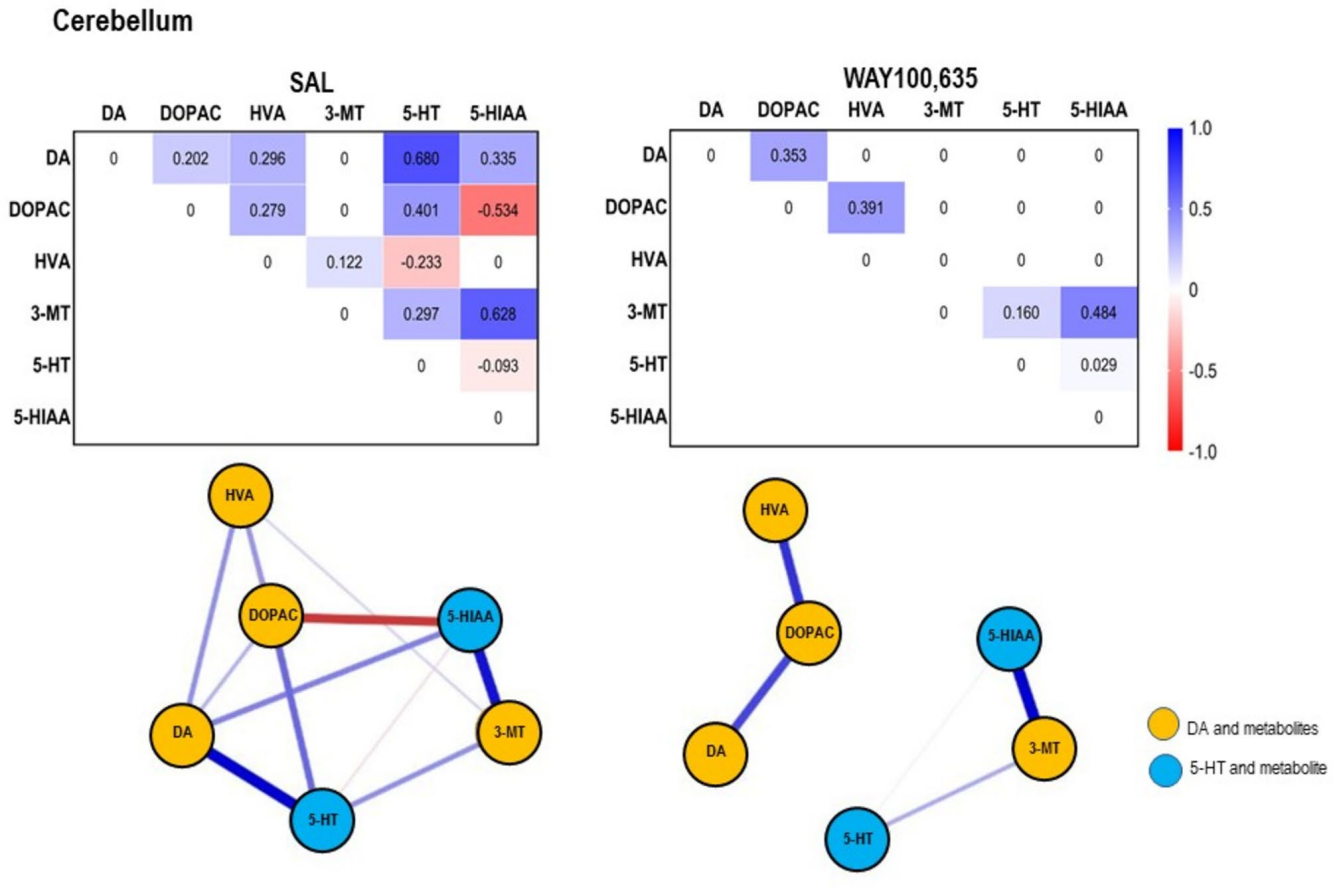
*Top*: Path coefficient matrix obtained for the **cerebellum** with EBICglasso modelling of neurotransmitter (DA, 5-HT) and metabolite (DOPAC, HVA, 3-MT, 5-HIAA) concentrations after treatment with SAL (12 out of 15 possible connections, sparsity: 0.200) and WAY100,635 (5 out of 15 possible connections, sparsity: 0.667). *Bottom*: Connections between DA, 5-HT and metabolites (DOPAC, 3-MT, HVA, 5-HIAA) in **cerebellum** after SAL and WAY100,635. Positive and negative associations are represented by blue and red lines, respectively. The thickness of the lines indicates the strength of the individual connections.

*Cingulate*. The analysis of HPLC data revealed an increase of HVA levels (Table. 1, Fig. 2B) in CING after WAY100,635 relative to SAL (p = 0.005; “treatment “: F_1,7,7_, 0.0176, p = 0.895; “brain region”: F_1,7,7_, 30.281, p < 0.001; “treatment” x “brain region”: F_1,7,7_, 30.281, p < 0.001).

Turnover of DA to DOPAC (Table. 2, Fig. 3A) was reduced after WAY100,635 compared to SAL (p ≤ 0.0001; “treatment”: F_1,7,7_, 3.508, p = 0.063, “brain region”: F_1,7,7_ = 13.087, p < 0.001, “treatment x brain region”: F_1,7,7_, 3.088, p = 0.006). Likewise, turnover of DA to 3-MT (Table. 2, Fig. 3B) was lower after WAY100,635 (p ≤ 0.0001; “treatment”: F_1,7,7_, 3.508, p = 0.063, “brain region”: F_1,7,7_, 13.087, p < 0.001, “treatment x brain region”: F_1,7,7_, 3.088, p = 0.006). 5-HIAA concentrations (Table. 1, Fig. 2D) were decreased after WAY100,635 (p ≤ 0.0001; “treatment”: F_1,7,7_, 6.751, p < 0.010; “brain region”: F_1,7,7_, 42.018, p < 0.001; “treatment x brain region”: F_1,7,7_, 2.943, p = 0.006).

In network analysis, after SAL, DA and DOPAC, and 5-HT and 5-HIAA showed strong negative connections with lower mean neurotransmitter than metabolite levels, indicating rapidly ongoing MAO-mediated degradation. DA and 3-MT exhibited a weaker positive association with low mean DA and high mean 3-MT, reflecting also active metabolization by COMT at the time of sacrifice. DOPAC and HVA were negatively associated with high DOPAC and low HVA, suggesting a delay in COMT-mediated HVA formation. 3-MT and HVA were positively associated, with the only slightly higher mean HVA level indicating slow but ongoing MAO-mediated degradation (Table. 1, Fig. 4).

After WAY100,635, the DA-DOPAC connection became positive, suggesting ongoing MAO-mediated conversion, while DA-3-MT, DOPAC-HVA, and 3-MT-HVA associations disappeared. Reduced DOPAC/DA and 3-MT/DA ratios together with higher DA and DOPAC levels relative to SAL indicate that initially high DA levels underwent rapid degradation by MAO, incurring also high levels of the primary metabolite. At sacrifice, the positive DA-DOPAC connection was still visible, but with a subdued turnover ratio likely due to saturation. An initially rapid and then abated degradation is also suggested by elevated HVA along with a decreased 3-MT/DA ratio relative to SAL. The increase of HVA reveals that the parallel steps of COMT- and MAO-mediated degradation had also taken place, but, at sacrifice, were not reflected any more by path coefficients between the individual nodes. This may be due to saturation effects at least in part of the animals, as reflected by the comparatively high interindividual variation. The negative 5-HT-5-HIAA association was lost, with the high 5-HIAA variability also suggesting suppressed MAO-mediated degradation at least in part of the animals, which is in line with the reduced 5-HIAA levels after WAY100,635.

*Caudateputamen*. DA concentrations (Table. 1, Fig. 2A) after WAY100,635 were higher relative to SAL (p ≤ 0.001; “treatment”: F_1,7,7_, 0.954, p = 0.330; “brain region”: F_1,7,7_, 12.084, p < 0.001; “treatment x brain region”: F_1,7,7_, 7.667, p < 0.001). Moreover, WAY100,635 decreased the turnover of 3-MT to HVA (Table. 2, Fig. 3B) relative to SAL (p = 0.002; “treatment”: F_1,7,7_, 0.599, p = 0.440, “brain region”: F_1,7,7_, 19.90, p < 0.001, “treatment x brain region”: F_1,7,7_, 1.748, p = 0.103).

After SAL, DA and DOPAC showed a weak positive association with higher mean DOPAC than DA, indicating ongoing MAO-mediated conversion. DA and 3-MT exhibited a stronger, weakly negative association with higher DA than 3-MT, suggesting delayed COMT-mediated turnover. DOPAC and HVA were positively associated with higher DOPAC than HVA, indicating decelerated but ongoing COMT-mediated conversion. No 3-MT-HVA connection was observed. The high HVA variability implies suppressed MAO-mediated degradation in some animals. 5-HT and 5-HIAA showed a weak positive connection with higher 5-HIAA than 5-HT, reflecting ongoing MAO-mediated conversion (Table. 1, Fig. 5).

After WAY100,635, DA-DOPAC and DA-3-MT associations strengthened and became positive, indicating ongoing MAO- and COMT-mediated conversion. DOPAC-HVA retained a strong positive connection, with the higher DOPAC than HVA level indicating a retardation of COMT-mediated turnover. The introduction of a strong, negative 3-MT-HVA connection with lower 3-MT than HVA indicates a predominance of MAO-mediated metabolization to HVA. The elevation of DA after WAY100,635 may reflect either initial DA increase with early saturation or a suppression or retardation of turnover. The positive 5-HT-5-HIAA connection was stronger, indicating enhanced MAO-mediated conversion.

*Nucleus accumbens*. After WAY100,635, DA levels (Table. 1, Fig. 2A) exceeded those obtained after SAL (p = 0.019; “treatment”: F_1,7,7_, 0.954, p = 0.330; “brain region”: F_1,7,7_, 12.084, p < 0.001; “treatment x brain region”: F_1,7,7_, 7.667, p < 0.001).

After SAL, DA was positively associated with DOPAC and 3-MT, and 5-HT with 5-HIAA, indicating ongoing MAO- and COMT-mediated DA degradation to DOPAC and 3-MT, respectively, and MAO-mediated 5-HT degradation to 5-HIAA. 3-MT was positively and DOPAC negatively associated with HVA with mean DOPAC higher and mean 3-MT lower than HVA, indicating ongoing MAO-mediated 3-MT-to-HVA but delayed COMT-mediated DOPAC-to-HVA conversion (Table. 1, Fig. 6).

After WAY100,635, a positive DA-HVA connection persisted, suggesting that prior metabolization steps had occurred, but were not captured by path coefficients for DA-DOPAC, DOPAC-HVA, DA-3-MT, and 3-MT-HVA at the time of sacrifice. Although mean DOPAC was high, so was the interindividual variation, which implies suppression of MAO-mediated degradation in some animals. The loss of DA-3-MT and DOPAC-HVA connections with higher DA and DOPAC than HVA, suggest reduced COMT- and MAO-mediated metabolism, contributing to the elevated DA levels compared to SAL. Elevated ventrostriatal DA after WAY100,635, may reflect either initial DA increase with subsequent saturation or a suppression or retardation of turnover. The positive 5-HT-5-HIAA association was lost, indicating inhibited MAO-mediated conversion.

*Thalamus.* DA concentration (Table. 1, Fig. 2A) after WAY100,635 was lower than after SAL (p ≤ 0.001; “treatment”: F_1,7,7_, 0.954, p = 0.330; “brain region”: F_1,7,7_, 12.084, p < 0.001; “treatment x brain region”: F_1,7,7_, 7.667, p < 0.001). Also, 5-HIAA levels (Table. 1, Fig. 2D) were diminished after WAY100,635 (p = 0.015; treatment”: F_1,7,7_, 6.751, p < 0.010; “brain region”: F_1,7,7_, 42.018, p < 0.001; “treatment x brain region”: F_1,7,7_, 2.943, p = 0.006).

After SAL, DA was positively associated with DOPAC but negatively and weaker with 3-MT, while 3-MT was positively associated with HVA. This indicates ongoing MAO- and COMT-mediated conversion of DA to DOPAC and 3-MT, respectively, with rapid, subsequent MAO-catalyzed turnover of 3-MT to HVA (as mean HVA exceeded mean 3-MT). 5-HT was positively associated with 5-HIAA, reflecting ongoing MAO-mediated 5-HT metabolization (Table. 1, Fig. 7).

After WAY100,635, DA-DOPAC and DOPAC-HVA (both positive) as well as DA-3-MT (negative) and 3-MT-HVA (positive) associations strengthened. This suggests, for one, ongoing MAO- and COMT-mediated DA degradation via the pathway DA → DOPAC → HVA. Higher DA than 3-MT and slightly higher HVA than 3-MT, however, indicate decelerated COMT-mediated DA-to-3-MT but accelerated MAO-mediated 3-MT-to-HVA conversion, which may explain the reduction in thalamic DA after WAY100,635. The 5-HT-5-HIAA association was slightly strengthened, although the decline in 5-HT concentration and turnover ratio may account for the decrease of 5-HIAA relative to SAL (Table. 1 and 2, Fig. 7).

*Dorsal and ventral hippocampus*. No differences in concentrations or turnover ratios were observed between WAY100,635 and SAL (Table. 1 and 2; 0.118 ≤ p ≤ 0.995).

After SAL, dHIPP showed no DA-DOPAC, but a weak positive DA-3-MT connection, suggesting low COMT-mediated DA degradation. Conversely, the vHIPP displayed a weak positive DA-DOPAC connection, indicating minor MAO-mediated DA degradation. In dHIPP, DOPAC-HVA was strongly positive with no 3-MT-HVA link, whereas, in vHIPP, DOPAC-HVA was weakly positive with a strong negative 3-MT-HVA connection, indicating a COMT-dominated second-step degradation in dHIPP and a MAO-dominated second-step degradation in vHIPP. In dHIPP, 5-HT was strongly positively associated with 5-HIAA, indicating MAO-mediated degradation, absent in vHIPP (Table. 1, Fig. 8).

After WAY100,635, no changes in metabolite concentrations or turnover ratios occurred in dHIPP or vHIPP versus SAL. In dHIPP, DA showed a weak negative DA-DOPAC and strong positive DA-3-MT connection; DOPAC-HVA weakened, and a negative 3-MT-HVA connection emerged. Mean DOPAC and 3-MT exceeded DA, but not HVA, indicating ongoing MAO- and COMT-mediated degradation with rapid further metabolization. The 5-HT-5-HIAA association in dHIPP slightly strengthened, reflecting ongoing MAO-mediated conversion. In vHIPP, no connections were detected. However, low DA, slightly higher DOPAC and 3-MT, and much higher HVA and 5-HIAA concentrations imply prior MAO- and COMT-mediated steps, not reflected any more by path coefficients at sacrifice (Table. 1, Fig. 8).

*Brainstem*. 5-HT levels (Fig. 2C) were lower after WAY100,635 compared to SAL (p = 0.035; “treatment”: F_1,7,7_, 2.004, p = 0.159, “brain region”: F_1,7,7_, 95.154, p < 0.001; “treatment x brain region: F_1,7,7_, 0.954, p = 0.467).

After SAL, DA showed no association with DOPAC, but a strong negative connection with 3-MT. No 3-MT-HVA link was detected, whereas DOPAC and HVA were negatively associated. Higher mean 3-MT than DA and lower DOPAC than HVA indicate rapid COMT-mediated steps of degradation. The 5-HT-5-HIAA connection was weakly positive, suggesting low but steady MAO-mediated conversion (Table. 1, Fig. 9).

After WAY100,635, a slight negative DA-DOPAC connection emerged, and the strong negative DA-3-MT association weakened. Lower mean DOPAC than DA and higher mean 3-MT reflect accelerated COMT-mediated turnover. No DOPAC-HVA or 3-MT-HVA connections were detected. Yet, higher mean HVA than DOPAC and 3-MT suggest prior MAO- and COMT-mediated degradation, with high variability obscuring path coefficients. The weak positive 5-HT-5-HIAA connection became strongly negative. The higher mean 5-HIAA than 5-HT levels indicate rapid MAO-mediated conversion, explaining reduced 5-HT levels compared to SAL (Table. 1, Fig. 9).

*Cerebellum*. The turnover ratio of DA to 3-MT (Fig. 3B) was increased after WAY100,635 relative to SAL (p = 0.013; “treatment”: F_1,7,7_, 3.508, p = 0.063, “brain region”: F_1,7,7_, 13.087, p < 0.001, “treatment x brain region”: F_1,7,7_, 3.088, p = 0.006).

After SAL, DA-DOPAC, DOPAC-HVA, and 3-MT-HVA were positively connected, indicating ongoing conversion. No DA-3-MT connection existed. Similar mean DA and DOPAC levels with DOPAC lower than HVA suggest a slowed MAO-mediated first step, but an accelerated COMT-mediated second step in degradation. Higher 3-MT than HVA indicates retarded MAO-mediated 3-MT-to-HVA conversion. A weak positive 5-HT-5-HIAA connection suggests low, but ongoing MAO-mediated conversion.

WAY100,635 strengthened the positive associations between DA and DOPAC and DOPAC and HVA. With mean DA lowest and mean HVA exceeding mean DOPAC, an accelerated degradation - firstly by MAO and subsequently by COMT - can be inferred. No DA-3-MT connection was observed, but the high mean 3-MT and the increased 3-MT/DA ratio suggest prior COMT-mediated DA metabolization, not reflected any more by path coefficients. The 5-HT-5-HIAA connection was abolished, indicating reduced MAO-mediated conversion (Table. 1,2, Fig. 10).

### Correlation between neurochemical and behavioral parameters

The significant positive and negative correlations after each treatment between the individual behavioral parameters in the test trial and the neurotransmitter and metabolite concentrations in the individual brain regions are given in Table. 3. Taken together, after SAL, correlations between behaviors and ventrostriatal as well as hippocampal neurotransmitter/metabolite levels were the most frequent. Associations were positive between rearing and grooming frequency and 5-HIAA as well as between rearing duration and 5-HT and HVA concentrations in NAC. Furthermore, duration and frequency of polyhedron exploration in the test trial were positively correlated with DA and 5-HT concentrations in this region. Also, positive correlations were observed between ambulation duration, grooming frequency and frequencies of polyhedron and cylinder exploration and 5-HT and/or 5-HIAA or HVA levels in dHIPP, as well as between duration and frequency of polyhedron exploration on DA levels and frequency of cylinder exploration and duration of rearing on 5-HIAA levels in vHIPP. Consistently, in all three regions, sitting duration and/or frequency were correlated with 5-HT and/or 5-HIAA concentrations.

**Table 3:** Correlations between the concentrations of dopamine (DA), serotonin (5-HT), 3, 4-dihydroxyphenylacetic acid (DOPAC), 3-methoxytyramine (3-MT), homovanillic acid (HVA) and 5-hydroxyindole-acetic acid (5-HIAA) in cingulate (CING), caudateputamen (CP), nucleus accumbens (NAC), thalamus (THAL), dorsal hippocampus (dHIPP), ventral hippocampus (vHIPP), brainstem (BS) and cerebellum (CER) and the following behavioral parameters assessed in the test trial after treatment with SAL (black) and WAY100,635 (green): overall activity (oac), ambulation duration (ambd), ambulation frequency (ambf), sitting duration (sittd), sitting frequency (sittf), sitting duration (sittd), sitting frequency (sittf), duration of head-shoulder motility (hsmd), frequency of head-shoulder motility (hsmf), grooming duration (groomd), grooming frequency (groomf), duration of polyhedron exploration (exppd), frequency of polyhedron exploration (exppf), duration of cylinder exploration (expcd) and frequency of cylinder exploration (expcf). Given are the Pearson product moment correlation coefficients (r) with p values ≤ 0.05.

	**oac**	**ambd**	**ambf**	**sittd**	**sittf**	**reard**	**rearf**	**hasmd**	**hasmf**	**groomd**	**groomf**	**exppd**	**exppf**	**expcd**	**expcf**
**CING** **DA** SAL WAY **DOPAC** SAL WAY **3-MT** SAL WAY **HVA** SAL WAY **5-HT** SAL WAY **5-HIAA** SAL WAY			-0.606	0.663			0.707			-0.708		-0.619	-0.671	-0.612	
**CP** **DA** SAL WAY **DOPAC** SAL WAY **3-MT** SAL WAY **HVA** SAL WAY **5-HT** SAL WAY **5-HIAA** SAL WAY	0.712 0.692 0.678	0.611	0.658 0.696		0.661		0.593			0.664				0.653 -0.701	
**NAC** **DA** SAL WAY **DOPAC** SAL WAY **3-MT** SAL WAY **HVA** SAL WAY **5-HT** SAL WAY **5-HIAA** SAL WAY					-0.679		0.707			0.626 0.659	0.742	0.826 0.781	0.728 0.760 0.667		
**THAL** **DA** SAL WAY **DOPAC** SAL WAY **3-MT** SAL WAY **HVA** SAL WAY **5-HT** SAL WAY **5-HIAA** SAL WAY		0.737 0.818	0.687	-0.742	-0.662		0.689				0.806				0.727
**dHIPP** **DA** SAL WAY **DOPAC** SAL WAY **3-MT** SAL WAY **HVA** SAL WAY **5-HT** SAL WAY **5-HIAA** SAL WAY	0.728	0.638 0.703	0.700 0.597	-0.664 -0.687	-0.687		0.680			-0.629	0.897 0.668 0.823		0.648 0.611 0.735		0.725 0.790
**vHIPP** **DA** SAL WAY **DOPAC** SAL WAY **3-MT** SAL WAY **HVA** SAL WAY **5-HT** SAL WAY **5-HIAA** SAL WAY			0.595	-0.728	-0.627	0.674 0.723	0.783 0.867			-0.649	-0.691	0.777	0.794		0.760
**BS** **DA** SAL WAY **DOPAC** SAL WAY **3-MT** SAL WAY **HVA** SAL WAY **5-HT** SAL WAY **5-HIAA** SAL WAY	0.673	0.620	0.633					0.706				0.693			
**CER** **DA** SAL WAY **DOPAC** SAL WAY **3-MT** SAL WAY **HVA** SAL WAY **5-HT** SAL WAY **5-HIAA** SAL WAY											0.859	-0.702 0.776	0.757		

After WAY100,635, correlations were most frequent between behavioral parameters and neurotransmitter/metabolite levels in CING, CP, dHIPP and BS. Associations were negative between ambulation duration and DA as well as between grooming frequency and DOPAC in CING. Moreover, duration and frequency of polyhedron as well as duration of cylinder exploration were negatively associated with DOPAC levels in CING. In turn, correlations were positive between overall activity and DOPAC, ambulation duration/ frequency and both DA and 5-HT, rearing frequency and 5-HT and grooming duration and DA in CP. Likewise, in the CP, overall activity, ambulation, rearing and grooming frequency as well as the frequency of polyhedron exploration correlated positively with either 3-MT, HVA or both, while, in BS, overall activity, ambulation duration and frequency, duration of head-shoulder motility and the duration of polyhedron exploration exhibited a positive correlation with DOPAC.

## Discussion

WAY100,635 increased DA levels in CP and NAC, and decreased DA levels in THAL. Moreover, WAY100,635 decreased 5-HT levels in BS. HVA concentrations were augmented in CING after WAY100,635, whereas 5-HIAA levels were decreased in both CING and THAL. Additionally, WAY100,635 reduced DOPAC/DA and 3-MT/DA turnover ratios in CING and HVA/3-MT turnover ratios in CP, whereas metabolization of DA to 3-MT was increased in CER.

WAY100,635 increased neostriatal DA levels, consistent with previous in vivo microdialysis^21^ and imaging^8^ studies. Also in line with previous microdialysis reports, no changes in 5-HT levels in NAC and HIPP^11^ were detected. The present results on ventrostriatal and thalamic DA, however, contradict former in vivo microdialysis^12,21,23^ and neuroimaging findings^8^. Post mortem HPLC and in vivo microdialysis or imaging findings are difficult to compare, since, in the, not only the DA levels in the synaptic cleft, but the summations of synaptic and intracellular DA were measured in the individual brain regions. Hence, while the total DA levels determined in the present study were not different from vehicle, synaptic DA levels may have been actually decreased. In turn, synaptic DA level may have remained unaltered, while the determined total neostriatal DA levels were elevated. Disagreements of this kind may be due to compensatory mechanisms such as the activation or inhibition of inhibitory D_2_ autoreceptors^31^ at the presynaptic terminals.

Correlations after SAL indicated positive associations between exploratory and grooming behavior as well as object/place recognition and mesolimbic monoamine levels (DA, 5-HT) and – mainly 5-HT turnover under normal conditions. After WAY, the majority of correlations, in addition to dHIPP, were observed in CING, CP and BS. Thereby, negative associations were introduced between motor behaviors as well as object/place recognition and DA turnover to DOPAC and HVA, while, in CP, dHIPP and BS, motor as well as exploratory behaviors and, solely, place recognition were positively associated with DA turnover to DOPAC, 3-MT and HVA.

WAY100,635 reduced overall activity and head-shoulder motility compared to SAL, while object and place recognition remained unaffected. This reduction in motor behavior is in line with findings from our earlier study^8^, whereas others reported no effect on ambulation^9–12^ and either an increase^8,13^ or no effect on exploratory parameters^9–12^. The lack of effect on object recognition concurs with Pitsikas et al.^14^, but contrasts with Pitsikas et al.^15^ and Carey et al.^16^. These discrepancies likely reflect differences in dose, timing, and behavioral paradigms: in our study, 0.3 mg/kg were administered post-sample trial, with a 5-min test 30 min later following habituation trials, while. previous studies varied in dose (1 mg/kg,^15^*, 2003*; 0.025–0.05 mg/kg,^16^), timing (pre- or post-sample trial), and procedures, such as the selection of longer intertrial intervals or repeated exposures to novel objects^15,16^).  Besides, open field test durations ranged from 5 to 90 min and were conducted with or without prior habituation^8–11,13^. Apart from this, however, both correlation and network analysis imply that the behavioral effects of WAY100,635 may be basically related to regional alterations of monoamine levels and turnover.

The locomotor circuitry is directly innervated by 5-HT neurons in the caudal BS and spinal cord^32–34^. 5-HT may have excitatory as well as inhibitory effects on spinal motoneurons (for review see^35^): while application of 5-HT to the dendrites increased excitability via 5-HT_2_R action, application to the soma inhibited motoneuron firing by 5-HT_1A_R activation. Thus, the systemic application of a 5-HT_1A_R antagonist such as WIN-100635 ought to have activated rather than inhibited motor behaviors at the spinal level. However, 5-HT_1A_R antagonism prevented 5-HT_1A_ autoreceptor-mediated inhibition of 5-HTergic neurons in the raphe nuclei, leading to an increase of 5-HT efflux (see, e.g.,^36^). Conversely, augmented 5-HT levels in the somata and terminal regions of the 5-HTergic-system activated inhibitory somatodendritic autoreceptors in the raphe nuclei, thereby again reducing the firing frequency of 5-HTergic neurons (see, e.g.,^37^). Hence, under the present experimental conditions, systemic WAY100,635, initially, may have blocked 5-HT_1A_ heteroreceptors at the level of the spinal cord and/or inhibited 5-HT_1A_ autoreceptors in the raphe nuclei, leading to an elevation of 5-HT. Then, two different mechanisms may have joined in action: firstly, the augmentation of 5-HT may have led to a renewed activation of inhibitory autoreceptors in the raphe nuclei, thereby again reducing the level of available 5-HT. Secondly, as revealed by network analysis, a strong association between 5-HT and 5-HIAA was introduced, which may be interpreted to reflect the promotion of (MAO-catalyzed) metabolic turnover. Both processes may account for the observed reduction of 5-HT concentrations in the BS, which, in turn, can explain the overall reduction of motor/exploratory activities.

The BS sends 5-HTergic efferents to the CING^38^, which contains a high amount of 5-HT_1A_R binding sites^39^. Selective 5-HT_1A_R agonists such as 8-OH-DPAT increase DA in the prefrontal cortex^40^. Hence, WAY100,635, arguably, not only decreased 5-HTergic input from the BS, but, additionally, blocked the DA efflux from the synaptic terminals of ascending DAergic neurons (e.g., from the ventral tegmental area [VTA];^41^) in the CING. The hypothesis of reduced 5-HTergic input is supported by the (compensatory) suppression of 5-HT degradation in the CING, which is in line with the observed decline of 5-HIAA after WAY100,635. There is evidence that, also in the CING, DA concentrations are regulated by presynaptic D_2_ autoreceptors at the presynaptic terminal^42,43^. From this may be inferred that the decline of DA levels incurred a compensatory increase of DA efflux, resulting in the enhancement of DA turnover via both the MAO- and the COMT-mediated pathways (as suggested by network analysis), and, ultimately, in the increased concentration of HVA after WAY100,635.

The CP receives ascending 5-HTergic fibers from the dorsal raphe nucleus^44^. In vitro studies with striatal preparations have either reported an inhibitory (e.g.,^45^) or an excitatory effect of 5-HT on neostriatal DA release (e.g.,^46^). As outlined above, an initial increase of 5-HT followed by a compensatory decrease may be surmised for the BS. Hence, a reduction of the inhibitory action exerted by the excitatory 5-HT_1A_R subtype can be inferred, which, apparently, was not entirely blunted by the administration of the 5-HT_1A_R antagonist. Since the CP is inhibited by γ-aminobutyric acid (GABA)ergic microcircuits^47^, it can be surmised that WAY100,635 disrupted the inhibition exerted by the GABAergic microcircuits in the CP, adding to the increase of available DA. Neostriatal DA concentrations are regulated by presynaptic D_2_ autoreceptors^31^. Remarkably, the WAY100,635-induced elevation of available DA was so high that the inhibitory feedback mechanism failed to normalize DA levels in the CP, at least in the present time window between administration and sacrifice. The increase of DA in the CP was associated with a promotion of MAO-catalyzed turnover. Likely, the elevation of DA occurred first in order, causing a saturation with respect to COMT-catalyzed degradation.

The raphe nuclei send 5-HTergic efferents to the VTA^48^, from which the NAC, in turn, receives DAergic projections^49^. 5-HT triggers DA release from somatodendrites and nerve terminals^50^*.* The observed decline of 5-HT in the BS, for one, may induce also a reduction of 5-HTergic input to the VTA and, thus, a decline of DA release in the NAC. However, the NAC also receives inhibitory GABAergic^51^ and excitatory GLUergic input^52^ from the neocortex. Since the 5-HT_1A_ heteroreceptor inhibits GABA as well as GLU release^53^, treatment with the 5-HT_1A_R antagonistic WAY100,635, likely, reversed these effects, resulting in a net increase of excitatory GLUergic input, which may have triggered the observed elevation of DA. Also here, however, the outcome of network analysis, reflecting a suppression of MAO- and COMT-catalyzed DA turnover, raises the question, whether WAY100,635 augmented the available DA to such an extent that a saturation was reached, or whether it, rather, suppressed DA turnover, leading to the observed elevation of DA.

Various thalamic nuclei express high densities of 5-HT_1A_R binding sites^54^. Moreover, thalamic nuclei receive GABAergic and GLUergic projections from basal ganglia^55^ and neocortex^56^*,* respectively. The 5-HT_1A_ heteroreceptor mediates inhibition of GABA release in the THAL^57^ and of GLU release in the neocortex^53^. Hence, the direct 5-HT_1A_ antagonistic effects of WAY100,635 may have led to an elevation of both thalamic GABA and neocortical GLU efflux. One explanation for the present findings may be that, under the present experimental conditions, the inhibitory GABAergic input outweighed the excitatory GLUergic input from the neocortex, resulting in the observed reduction of thalamic DA after WAY100,635. On the other hand, the WAY-induced promotion of DA turnover via both the MAO- and the COMT-catalyzed pathways, rather suggests a first-in-order overweight of excitatory GLUergic input from the neocortex, with the resulting increase of DA incurring an elevation of DA catabolism as an adaptive response.

The CER receives 5-HTergic afferents from various reticular and raphe nuclei^58^ and DAergic afferents mainly from the VTA^48^. A decrease of 5-HT in the BS, thus, likely also incurs a decrease of available 5-HT in the CER. This assumption is corroborated by the (compensatory) suppression of 5-HT metabolization indicated by network analysis. Since the VTA also receives 5-HTergic fibers from the raphe nuclei^48^, the observed decline of 5-HT in the BS, for one, can be assumed to reduce 5-HTergic input to the VTA and, subsequently, also the concentration of DA in the CER. On the other hand, 5-HT blocks excitation induced by GLU or aspartate as well as inhibition by GABA via 5-HT_1A_R action^59^. Hence, the antagonistic action of WAY100,635 may have incurred a net excitation by GLU, resulting in an elevation of DA levels beyond the decrease due to the reduction of 5-HTergic input from the BS. This assumption is consistent with the enhancement of (compensatory) DA turnover via both the MAO- and the COMT-mediated pathway suggested by network analysis.

WAY100,635 primarily affected the CING, where increased DA turnover -likely compensatory to reduced DA availability - was observed. The anterior and posterior CING are involved in object and spatial processing, respectively^60,61^. Thus, disruption of DA balance in this region may underlie the absence of effects on recognition memory. Additional DA alterations in CP, NAC, THAL, and CER - regions also implicated in recognition memory^27^ - may have contributed to this outcome.

In the present study, the 5-HT_1A_R antagonistic treatment induced widespread effects on neurotransmitter and metabolite levels, as well as turnover ratios. Together with the outcome of network analyses, these results suggest a promotion of MAO-catalyzed DA turnover in dHIPP, COMT-catalyzed DA turnover in vHIPP and BS and both MAO- and COMT-catalyzed DA turnover in CING, CP, THAL and CER. In contrast, both MAO- and COMT-catalyzed DA turnover was suppressed in NAC. Moreover, WAY100,635 promoted (MAO-catalyzed) 5-HT turnover in CING, CP, BS, dHIPP and CER, but suppressed it in NAC and THAL. Thus, while the 5-HT_2A_R antagonist ALT had suppressed DA metabolization by both MAO and COMT in the THAL^24^, the 5-HT_1A_R antagonist WAY100,635 suppressed DA metabolization by both MAO and COMT in the mesolimbic NAC. Both ALT and WAY100,635 promoted DA metabolization by MAO in CING, dHIPP and CER. However, while ALT had facilitated DA metabolization by MAO in the mesolimbic NAC, it was facilitated by WAY100,635 also in key regions of the nigrostriatal system (CP, THAL). Furthermore, both ALT and WAY100,635 promoted DA metabolization by COMT in regions of the nigrostriatal (CP) and mesolimbic system (CING, vHIPP) as well as in CER. However, while ALT had facilitated DA metabolization by COMT in NAC, it was facilitated by WAY100,635 in THAL and BS. After both ALT and WAY 100,635, 5-HT metabolization (by MAO) was suppressed in THAL; WAY100,635, however, added a suppression in NAC. Moreover, ALT promoted 5-HT metabolization merely in dHIPP, whereas, under WAY100,635, 5-HT metabolization was additionally promoted in CING, CP, BS and CER. Thus, taken together, 5-HT_2A_R antagonism preferentially affected mesolimbic circuits, while 5-HT_1A_R antagonism modulated both mesolimbothalamic and nigrostriatal pathways. Importantly, the suppression of DA and 5-HT turnover in NAC under WAY100,635 may reflect the role of the 5-HT_1A_R as an integrator of affective and cognitive processing^62^ and its centrality in motivation and reward^63^.

The observed region- and pathway-specific patterns underscore the functional complexity of 5-HT_1A_R signaling. The differential modulation of MAO and COMT pathways by WAY100,635 may help to explain its relevance in psychiatric conditions such as major depression and anxiety, supporting the therapeutic efficacy of 5-HT_1A_R agonists like buspirone and vortioxetine^64,65^. Future studies should further investigate regional differences in enzyme expression and kinetics to better understand how 5-HT_1A_R antagonistic activity shifts the predominance of metabolic pathways and, thus, may contribute to the pathophysiology underlying neuropsychiatric disorders. Besides, the present studies had been confined to male rats. Since it is known that estrous hormones increase not only the density of the 5-HT_1_R subtype in various brain regions including preoptic area, hypothalamus and septum^66^, but also regional levels of monoamines including 5-HT, DOPAC and norepinephrine^67,68^, future investigations should also include female rats, especially given the known female bias in the occurrence of neuropsychiatric disorders (for review see^69^).

## Conclusion

The 5-HT_1A_R antagonist WAY100,635 decreased overall activity and exploration and altered the quantitative relations between the neurotransmitter/metabolite levels in the individual brain regions, by inducing region-specific shifts in the metabolization pathways. Findings may be relevant for understanding the neurochemistry of DAergic and/or 5-HTergic dysfunction in psychiatric conditions.

## Data Availability

The original data are available from the corresponding author upon request.
